# Microtubules in Martini: Parameterizing a heterogeneous elastic-network towards a mechanically accurate microtubule

**DOI:** 10.1093/pnasnexus/pgaf202

**Published:** 2025-06-21

**Authors:** Abhilash Sahoo, Sonya M Hanson

**Affiliations:** Center for Computational Biology, Flatiron Institute, New York, NY 10010, USA; Center for Computational Mathematics, Flatiron Institute, New York, NY 10010, USA; Center for Computational Biology, Flatiron Institute, New York, NY 10010, USA; Center for Computational Mathematics, Flatiron Institute, New York, NY 10010, USA

**Keywords:** coarse-grained, Martini, microtubules, molecular dynamics, cytoskeletal modeling

## Abstract

Microtubules are essential cytoskeletal filaments involved in cell motility, division, and intracellular transport, exhibiting complex structural dynamics governed by diverse biophysical factors. Atomistic simulations of microtubule assemblies remain challenging due to their extensive spatiotemporal scales. To address this, we present a multiscale approach combining the primarily top-down Martini 3 coarse-grained (CG) model with an appropriately parameterized heterogeneous elastic network to capture microtubule mechanics and molecular detail efficiently. By iteratively tuning the elastic network, we matched the structural fluctuations of CG heterodimeric building blocks to atomistic reference data, reproducing experimentally consistent mechanical properties. This framework helped us identify stabilizing long-lived interactions between charged C-terminal tails and the folded domain of neighboring tubulin subunits, offering insight into sequence-specific contributions to lattice stability. Our efforts culminated in the construction of a ∼200 nm microtubule composed of ∼6 million interaction centers, enabling exploration of large-scale microtubule-associated processes with amino acid-level resolution. This work bridges the gap between molecular specificity and computational scalability, offering a platform for simulating biophysical processes across cellular length and time scales.

Significance Statement Microtubules are the primary structural components within cells responsible for various cellular functions. But due to their large size, their intricate dynamics is difficult to computationally model accurately with microscopic level structural information preserved. In this work, we successfully mimic the structural dynamics of microtubules by employing a primarily top-down coarse-grained model coupled with a parametrized heterogeneous elastic network. Balancing computational efficiency with molecular detail, the model revealed specific interactions driven by the disordered tubulin tails that influence microtubule stability, while also capturing accurate mechanical properties and structural behaviors at microtubule tips. This method opens new avenues for investigating cellular biophysics providing invaluable insights into microtubule-associated processes such as cell motility and transport at extended spatiotemporal scales.

## Introduction

Microtubules are a dynamic and complex assembly of proteins, that form an essential component of the cellular cytoskeleton. These microscopic cylindrical structures along with other interaction partners contribute to an array of critical physiological functions within the cell such as maintaining cellular structure, supporting cell division, and facilitating intracellular transport (1, 2). Microtubules are an assembly of tubulin heterodimer subunits—α/β-tubulin dimers that can rapidly assemble and disassemble, allowing cells to adapt to changing conditions and perform a wide array of dynamic functions (3, 4). Structurally, these tubulins can polymerize linearly creating protofilaments, which in turn can assemble into thin, hollow, cylindrical structures, with 11–16 protofilaments—with 13 being predominant in mammalian cells and 14 predominant in vitro. While *α*-tubulins exclusively bind to guanosine-5'-triphosphate (GTP), *β*-tubulins can exist in both GTP-bound and guanosine diphosphate (GDP)-bound states in vivo. The conformation of tubulin dimers, often elucidated through cryo-electron microscopy (cryo-EM) and X-ray crystallography, can vary based on their nucleotide states, associated binding partners and their relative position on the microtubule-lattice, leading to distinct structural and dynamical features in the resulting microtubule assembly (5–8). Conformational variances of individual tubulin dimers such as bending, twisting and/or fluctuations, coupled with induced cooperativity of the ordered microtubule lattices often results in assemblies that can respond to a variety of environmental stimulus thereby allowing reorganization of cytoskeletal architecture in response. For example, a dominantly GDP-tubulin lattice with a stabilizing GTP cap promotes microtubule stability and growth, whereas, a GDP only lattice promotes instability and disassembly. Considering this strong coupling between tubulin and microtubule dynamics, it is necessary to build an accurate picture of microtubules, with accounting for variances in tubulin dynamics, particularly with such mixed lattices in physiological microtubules.

Beyond structural studies, recent experiments have reported how environmental stimulus—buffer conditions and macromolecular crowding can influence microtubule biophysics such as mechanical properties, crowder-induced bundling, and the interaction between microtubules (7, 9–12). To supplement the molecular resolution detail of structure determination and the experiments that give access to macroscopic biophysical properties, but can lack mechanistic insight, computational and theoretical tools have been employed to develop a more comprehensive of microtubule biophysics. Examples of simple and intuitive in silico models of microtubules range from mean field approaches that do not explicitly incorporate detailed structural data to rule-based stochastic methods built with structural constraints from experimentally resolved structures (13–15).

However, a computational approach that can bridge the gap between structure determination and biophysical properties of microtubules is molecular dynamics (MD) simulations (16). While MD can be a powerful computational technique to model complex structural assemblies with full atomic-level information, the length scales required to model a full microtubule can make the method computationally prohibitive. Already, the structural characterization of tubulin dimers and smaller microtubule patches has been extensively studied with molecular simulations (17–21). Applications of All-Atom scale MD (AA-MD) is limited here to studies of dimers, dimer assemblies and smaller patches, <30–50 nm long (22). To our knowledge the largest relevant AA-MD study consists of a 14 protofilament microtubule with 6 dimers per protofilament, aiming to compare the dynamics of microtubule tips at different nucleotide associated states (20). Moreover, due to length-scale limitations it is generally not possible to study mixed lattices, often observed for stable and growing physiological microtubules—generally a GDP lattice with a GTP cap. An appealing alternative approach however is coarse-grained molecular dynamics (CG-MD) simulations, developed to bypass the computational bottleneck of AA-MD by reducing the resolution and locally averaging structural and dynamical information (23). Coarse-grained dynamics are typically faster due to the smoothing of the energy landscape and the removal of high-frequency atomistic degrees of freedom. Additionally, the loss of frictional and memory effects leads to reduced dissipation, resulting in accelerated motion (24–27).

Modeling efforts along this vein aim to build larger microtubule structures from atomic-level information, following a bottom-up coarse-graining approach, and allow bridging scales closer to experimental levels. This approach focuses on deriving simplified models (coarse-grained potentials) for complex molecular systems by preserving key structural and dynamical features as observed in atomistic simulations, typically through statistical mechanics-based techniques like the iterative Boltzmann inversion or force-matching (27–29). This approach enables efficient simulations of larger spatiotemporal scales, bridging molecular details with emergent behavior while balancing accuracy and computational cost. For example, Kononova et al. (30) built a bottom-up C*α*-based self organizing polymer (SOP) model with parameters derived from implicit solvent molecular dynamics with CHARMM19 force-field. This work established an in silico nano-indentation method with results comparable to experimental force-deformation spectra and also revealed free energies of tubulin–tubulin interactions—both lateral and longitudinal. Another approach by Deriu et al. involved using molecular dynamics to refine protein conformations and normal mode analysis to extract vibrational characteristics, which was subsequently used to create a coarse-grained model (31). Finally researchers also have developed ultracoarse-grained (UCG) modeling approaches with state-dependent potentials, and tailored elastic networks to probe large-scale motions without sacrificing essential dynamics. These systems have been applied to study filament mechanics, twist, and interactions with Taxol and Dynein over micrometer scales (32–36). However, these methods rely on explicit parametrization of the whole system, limiting their usage in other physiological contexts beyond the direct setup or the problem it was parameterized for. Moreover, considering the importance of local cellular environment in governing microtubule biophysics, there is a need for an explicit representation of local environment in the modeling strategies—often absent in the current coarse-grained approaches (11, 12).

To address this gap, in this work we propose a coarse-grained biomolecular model for microtubules built with a commonly used and general coarse-grained forcefield—Martini 3 (37). Martini is an intermediate-level coarse-grained model with 1–5 interaction sites representing each amino acid. This approach reduces computational demands while retaining essential structural and dynamic information. Martini has gained popularity for simulating large and complex biological systems, such as virions and organelles (38–41). Here, we report specific modifications to the elastic network architecture with the Martini 3 forcefield through an iterative approach. Our refined intermediate-resolution coarse-grained model of the microtubule demonstrates the capability to map structural and dynamical data from accurate all-atom simulations to larger scale structures, while preserving the transferability of the forcefield. In this work, we have tested this approach on GDP-associated microtubule lattice, with a GMPCPP cap (a non hydrolyzable GTP analog in lieu of GTP), and discussed the initial structural insights from this strategy. To our knowledge, this is the first hybrid coarse-grained molecular dynamics simulation of a complete microtubule, constructed with a mixed nucleotide lattice.

## Results

### An initial coarse-grained microtubule renders unstable

In the following sections, we discuss the modifications we employed to the Martini 3 forcefield towards creating a stable coarse-grained simulation of this microtubule, with focus on the GMPCPP microtubule caps.

#### Building a complete microtubule lattice

**Fig. 1.**
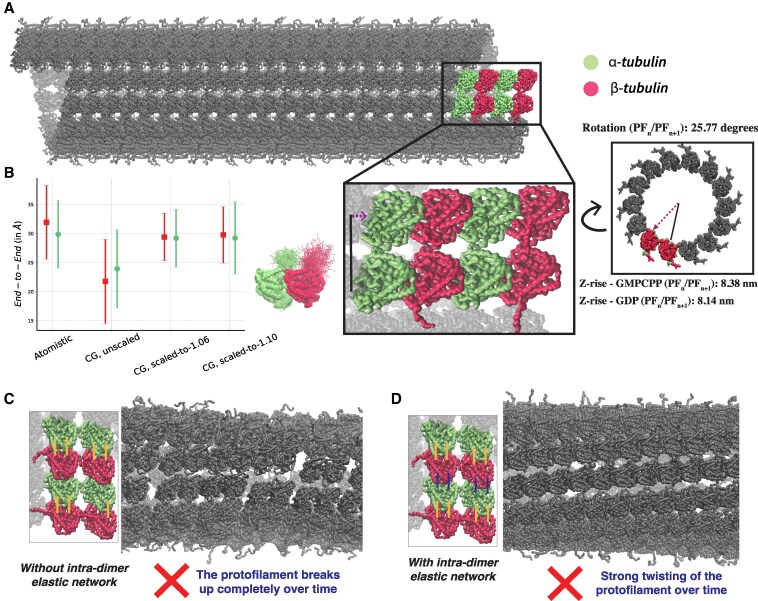
An initial CG microtubule. A) Model CG microtubule (14-3) assembled from translating and rotating cryo-EM structure (6DPV). B) End-to-End (ee) distance of tubulin tails, compared across atomistic, original-unscaled Martini forcefield and the Martini forcefield with scaled protein–water interactions. C) Out-of-box Martini 3 simulation with a constant elastic network across a single dimer only, D) across the entire protofilament.

Since microtubule structure and dynamics involves a complex interplay of tubulin dimers in various associated-nucleotide states, it is important to carefully prepare the initial lattice architecture. Towards creating our Martini microtubule, we built our primary lattice according to the cryo-EM structure of undecorated microtubule with GDP-tubulin lattice (6DPV) (42). We extrapolated the microtubule’s inherent helical twist (Rotation—${\mathrm{PF}}_{n}/{\mathrm{PF}}_{n+1}$, 0.874 nm) and axial rise (Z-rise—${\mathrm{PF}}_{n}/{\mathrm{PF}}_{n+1}$, $25.77^{\circ }$) from these segments to synthesize a single turn of the 14 protofilament-microtubule (Fig. 1A). Thereafter, these individual helical fragments were translated along the microtubule’s axis to create the initial microtubule structure with ∼19 dimers per protofilament. This initial structure was then capped with a six dimer-long GMPCPP-stabilized lattice structure (6DPU), with extrapolated helical twist (Rotation—${\mathrm{PF}}_{n}/{\mathrm{PF}}_{n+1}$, 0.874 nm) and axial rise (Z-rise—${\mathrm{PF}}_{n}/{\mathrm{PF}}_{n+1}$, $25.77^{\circ }$). Note that the microtubule seam in this context was deduced through rotations and translations of protofilaments, so any potential distinct conformational variation of the tubulins at the seam is not present. We used the *martinize* protocol to map the all-atom microtubule structures to the coarse-grained Martini 3 representation (43).

#### Disordered C-terminal tails

One advantage of using the Martini coarse-grained model is that residue-level detail is preserved while achieving a computational speed up. For example, both *α*-tubulins and *β*-tubulins feature disordered and highly charged C-terminal tails 19 and 18 amino acid long respectively, that can modulate their interactions with ions, metabolites, membranes, cytoskeletal assemblies, and microtubule-associated proteins (MAPs) (44–47). Previous studies have highlighted several alterations in the biophysical features and function of microtubules in the presence of intact C-terminal tails compared to those with truncated tails (48, 49). Therefore, it is necessary to model the C-terminal tails accurately, to allow this model to be extended to studies with a broader physiological focus. However, a prevalent challenge associated with Martini, is its tendency to promote more compact structural conformations, particularly in disordered regions. To mitigate this issue, a commonly employed strategy in both coarse-grained and atomistic forcefield development involves amplifying the interaction strength between protein and water.

Here, we adhered to the approach developed by Thomasen et al. (50), wherein the authors proposed an ad hoc range of scaling factors for protein–water interactions from 6% to 10% to effectively reproduce small-angle X-ray scattering (SAXS) data for multidomain proteins. This scaling factor was adopted to counterbalance the compacting effects of the forcefield and improve the accuracy of our simulations. For example, this scaling of 6% was recently used by Jussupow et al. (51) towards studying conformational states that govern Hsp90 conformational dynamics. To validate these modifications, we simulated a single GDP tubulin at atomistic resolution and compared against the mapped coarse-grained simulations, with the unmodified forcefield and the forcefield with up-scaled interaction by 6% and 10%. We used the end-to-end distance (ee) to validate and compare structural ensembles in our simulations. While both the radius of gyration ($R_{g}$) and ee are commonly used metrics for analyzing disordered regions, $R_{g}$ tends to be more error-prone for shorter polymer lengths (52, 53). In our results, the default Martini 3.0 forcefield produced more compact conformations, as indicated by smaller ee values. However, applying 6% and 10% up-scaling to the protein–water interactions resulted in structural ensembles that more closely matched atomistic values. In line with Thomasen et al.’s recommendation for multidomain proteins—and to minimize deviations from the validated Martini framework—we adopted a conservative 6% scaling in our simulations.

#### Out-of-the-box Martini results in broken/twisted microtubule lattice

Since the Martini forcefield is limited in its ability to accurately capture the details of highly charged groups and small molecules such as the nucleotides like GTP, GMPCPP, and GDP, we decided not to explicitly include these nucleotides, but rather encode the structural and dynamical aspects of the nucleotide presence through a carefully designed heterogeneous elastic network model (ENM). Since the tubulin dimers constitute the fundamental units of microtubules, we initially applied an elastic network with a constant strength on the tubulin–dimers only. This preserves the initial GDP-tubulin structure and relies on the protein–protein interactions from the Martini 3 forcefield to stabilize the overall lattice assembly. This ENM was designed following standard recommendations with *martinize* (54, 55), with interaction sites defined as *in-contact*, if the distance between them ranged from 5 Å to 9 Å; and a spring constant of 500 kJ/mol. Also, as noted, since the C-terminal tails are known to be disordered, we excluded the them from the elastic-network. We ran 400 ns of this initial out-of-the-box CG microtubule and found that the microtubule lattice fell apart rapidly, resulting in a loss of most intradimer contacts by the last 200 ns (Figs. 1C *top* and S1). This is probably due to the unstable protein–protein contacts at the interdimer interface resulting from the absence of explicit nucleotides to stabilize them.

In the next iteration, we extended the ENM to include interdimer contacts, along a single protofilament, to maintain interdimer contacts and the fidelity of the microtubule lattice structure. With the new extended ENM, the dimer–dimer interactions were preserved by the network, preventing breaking up of the lattice (Fig. 1C and D). However, the overall microtubule architecture started continuously twisting to maximize interprotofilament contacts (Fig. 1D).

Although, this twisting could in theory be solved by extending the ENM to interprotofilament contacts, we have avoided this approach in our systems as interprotofilament contacts are much weaker than intraprotofilament contacts and are not explicitly modulated by interactions with nucleotides/ligands. Moreover, not encoding the interprotofilament contacts through an elastic network also allows extending these systems to studies involving microtubule tips, mechanical deformations, and lattice defects.

To remove systematic effects that might arise due to the imbalance in interprotofilament interactions at the tips, particularly near the microtubule seam, and create a more biologically relevant microtubule patch, we randomized the length of the protofilament by removing tubulin dimers at both the ends.

### Systematic optimization of the ENM

Here, we hypothesize that scaling down the elastic network strength would allow more microscopic local fluctuations and reduce the systematic twisting of the microtubule. But instead of an explicit scaling, with a refined heterogeneous elastic network, we can appropriately encode these local fluctuations from an atomistic representation onto our coarse-grained forcefield. In this work, we aim to parametrize a corrective harmonic term through an iterative approach to the original Martini Hamiltonian to generate an effective Hamiltonian: $H_{\mathrm{eff}}=H_{\mathrm{Martini}}+H_{\mathrm{hENM}}$.

**Fig. 2.**
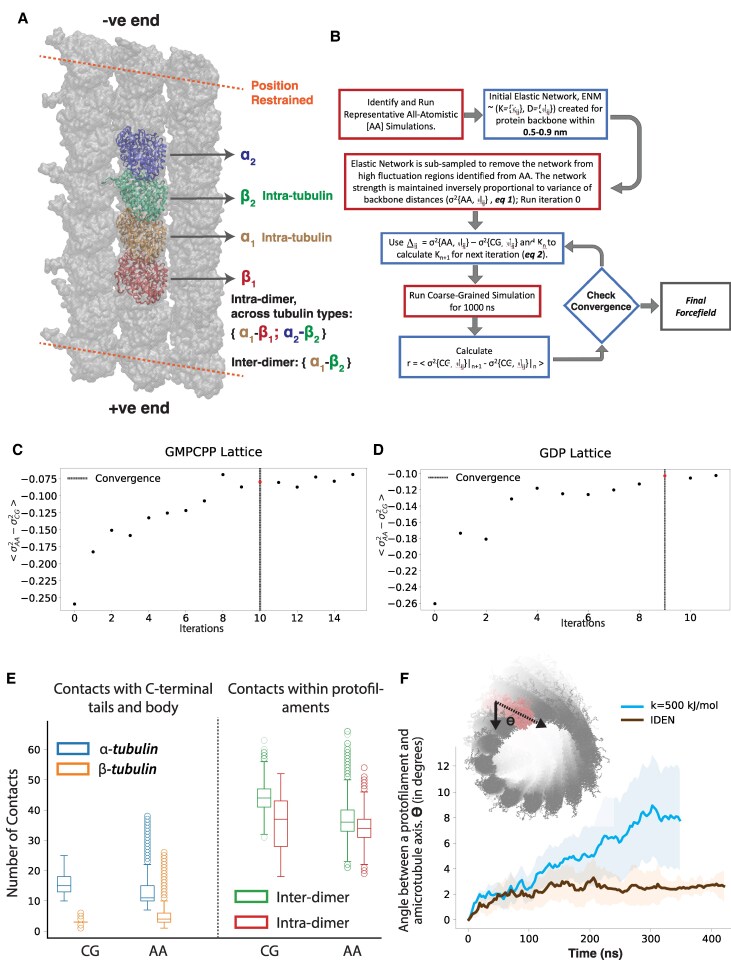
Parameterizing a more stable microtubule. A) The all-atom microtubule patch used to parametrize the CG ENM. The tubulin monomers used for mapping fluctuations are highlighted. B) Flowchart detailing steps for iterative optimization of elastic network. C, D) Average deviation in fluctuation between coarse-grained and all-atom simulations over iterations. C) GDP-lattice, D) GMPCPP-lattice. The dotted black lines indicate convergence of the iterative process. E) Comparisons of the number of contacts across AA (All-Atom) and CG simulations. On the left are comparisons between the C-terminal tail and the body of the tubulin. On the right are comparisons between interdimer and intradimer contacts within a single protofilament. F) Protofilament twisting in IDEN compared to simulations with a constant elastic network.

Our approach, with some careful alterations, closely follows the approach outlined by Globisch et al. (56), for capturing accurate structure and dynamics of the Cowpea Chlorotic Mottle Virus (CCMV) (Fig. 2B). First, we created an all-atom microtubule patch of three protofilaments, with four dimers each, built as previously described. The tubulins at the ends of the protofilament were center-of-mass position restrained (10 kJ/mol) to simulate conditions within the microtubule lattice (Fig. 2A). The new ENM thus has variable network strength based on the all-atom simulations, characterized by a set of spring constants ($K=\{k_{ij}\}$) and the corresponding equilibrium lengths ($D=\{d_{ij}\}$).

#### Inferring the heterogeneous elastic network with an iteratively refined distance-based approach

The protocol [Iteratively refined distance-based elastic network (IDEN)] encodes the dynamics of the microtubule patch into the elastic network in two steps—first, by removing highly dynamic regions from the ENM, and second, by iteratively calibrating the ENM through appropriately scaling the elastic network (Fig. 2A–D). Please refer to the Methods section for details of this parameterization, and the Section S1 (Figs. S2 and S3) for a discussion on convergence.

This optimization protocol reduced the number of harmonic restraints for the GDP lattice by 7.45% for interdimer contacts (average spring constant 119.8 kJ/mol); and 7.8% for intradimer contacts (average spring constant 94.6 kJ/mol). Similarly for the GMPCPP lattice at the microtubule cap, the number of restrains reduced by 7.3% for interdimer contacts (average spring constant 125.4 kJ/mol); and 7.8% for intradimer contacts (average spring constant 93.1 kJ/mol). This allowed for some local frustration to be relieved in this large assembled structure, leading to an overall more stable microtubule model.

### Structural validations

In this section, we present a set of local structural validations of our coarse-grained microtubule model by comparing structural features from the full coarse-grained microtubule simulations with the atomistic simulations of the three-protofilament microtubule patch that was used for parametrizing our elastic network. Since, position restraints were applied to simulate a stable microtubule in atomistic simulations, we used the farthest tubulin dimer from the ends for our analysis to minimize the effects of these restraints (Fig. 2A). Considering the variations introduced in these structural analysis for the GMPCPP lattice due to their location at the tip, we compared the local structural details for the GDP-lattice only. The last 100 ns of all-atom (AA) simulations were used for analysis and was compared with last 500 ns of CG simulation, aggregated across three independent replicas. For the CG microtubule, only the central dimers from protofilament numbers 4 to 10 are used to minimize the effects that microtubule tips and seam might impart. Finally for the contacts at the microtubule seam, we used the central dimers of protofilament number 1 and 14, which constitute the microtubule seam.

Recent work have reported that interaction between C-terminal tails and the tubulin body can have both structural and functional relevance (48). Here we quantified the number of contacts between the tubulin tails and body (Fig. 2E). Interestingly, the number of tail-body contacts is significantly (about threefold) higher for *α*-tubulin compared to *β*-tubulins even though the number of residues on C-terminus considered for this analysis was equivalent for both tubulins.

Along a single protofilament, the number of interdimer contacts were higher than intradimer contacts. This could be due to discrepancy between the nucleotide states at both the interfaces in GDP microtubules. These trends for the tail-body contact and tubulin dimer contacts were replicated in our coarse-grained simulations as well. Now, across protofilaments, while highly dynamic, we found an overall higher number of contacts for *α*-tubulins compared to *β*-tubulins (Fig. S1). Since the number of tubulin contacts across neighboring protofilaments were varied and depended on their relative position across protofilament number and distance from the ends, we have not reported the corresponding for our coarse-grained simulations. Finally, the cross protofilament contacts at the seam follows the same trend, with more *α*-tubulin contacts compared to *β*-tubulin.

Finally, we quantified by the angle that a protofilament makes with the microtubule axis (Fig. 2F) to demonstrate a significantly reduced protofilament twisting due to IDEN. We note here that a some twist in this overall assembly is expected due to smaller associated length scales and finite sizes, similar to previous work with cellulose (57).

### Bio-mechanical validations—axial bending modulus

Microtubules are among the primary structural elements of the cell and their mechanical properties have been extensively investigated—both in isolation and in presence of physiological/simulated stimulus (11, 12, 58). For example, bending modulus has been measured either by directly pulling on the microtubules with optical tweezers or through fitting equilibrium thermal fluctuations to mathematical models (both linear and nonlinear) (12, 59). These studies have resulted in a range of reported values spanning about an order of magnitude depending on nucleotide and/or other ligand associated states. But the consensus observation is that the microtubules are highly rigid biological assemblies that are capable of withstanding extensive mechanical stress and remodeling other cellular assemblies like membranes.

Therefore, in order to extend our structural models to more complex physiological settings we need to be able to reproduce these elastic features. In this work, we measured flexural rigidity of the coarse-grained microtubules by two methods.

**Fig. 3.**
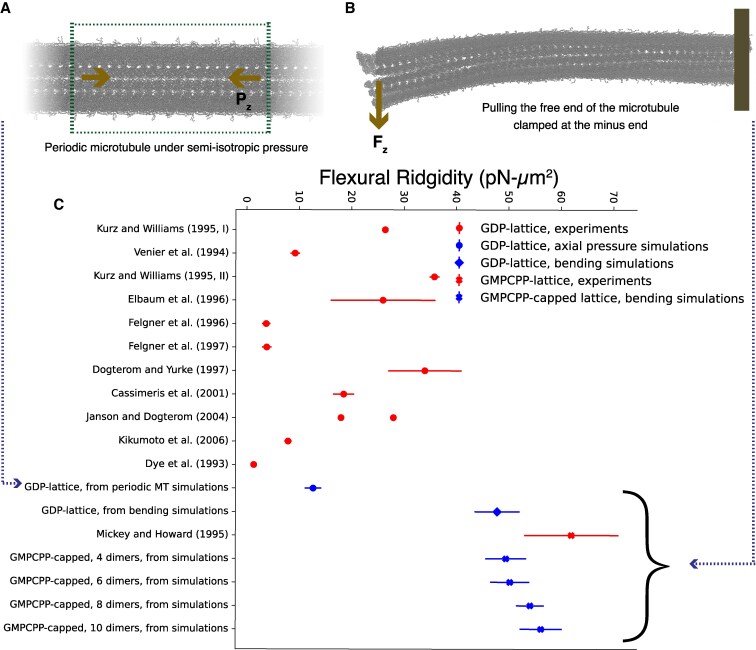
Simulating a microtubule that interacts with its periodic image establishes this microtubule exhibits mechanically correct properties. A) A representative snapshot of a microtubule under applied axial pressure (1 bar). B) Bending the microtubule from the plus-end that is position-restrained at the minus-end with steered molecular dynamics. C) Comparison of Flexural Rigidity from experiments and simulations. Experimental measurements are curated from Memet et al. (12). The results from experiments match with the corresponding simulations.

The first method involved creating a periodic microtubule (∼10 tubulin dimers long) along the microtubule axis and simulating it with applied anisotropic pressure along the axis ($P_{z}$, Fig. 3A). The slope of straight-line fit, assuming an elastic regime, in our inferred strain–strain plot resulted in $E_{a}$ = 0.64±0.08 GPa. Further, flexural rigidity ($\gamma =E_{a}I$) can be inferred using the second moment of cross-sectional area (I). Our simulations suggest a flexural rigidity of $12.6\;\mathrm{pN}\;\mu m^{2}$. These reported values are close to experimental reports and within the reported ranges (12, 59). Here, we want to highlight that this method assumes that microtubules are uniform and isotropic thin cylinders. It is difficult to assess the validity of this assumption and its impact on the microtubule mechanics.

Therefore considering the length scales of our CG microtubules, we employed a direct steering (at constant velocity) at plus end to estimate flexural rigidity using Euler–Bernoulli beam theory. The maximum deformation in our simulations was limited to 15 nm. We have used two different steering rates (1 and 2 nm/ns) to test the applicability of Euler–Bernoulli beam theory (*P*-value < 0.01 across both the rates), and report errors in our calculation of flexural rigidity. For GDP-only lattice, we report flexural rigidity of $45\;\mathrm{pN}\;\mu m^{2}$, which is a bit higher from our previous method. This could be due to assumptions encoded in inferring $E_{a}$ in both these approaches. For example with this second approach, errors could have also accumulated from the elastic-network preventing conformational changes in tubulins that might have happened over the course of the pulling simulations. But within the current framework, our reported values are very close to reports from a wide array of experiments. Another interesting observation here was with progressively increasing length of GMPCPP cap, the flexural rigidity increased, in line with experiments. This is in line with the previous work by Mickey and Howard (60) that suggested a significant increase in rigidity with GMPCPP microtubules compared to GTP and GDP ones.

### Structural analysis of GDP-bound microtubules with optimized ENM

**Fig. 4.**
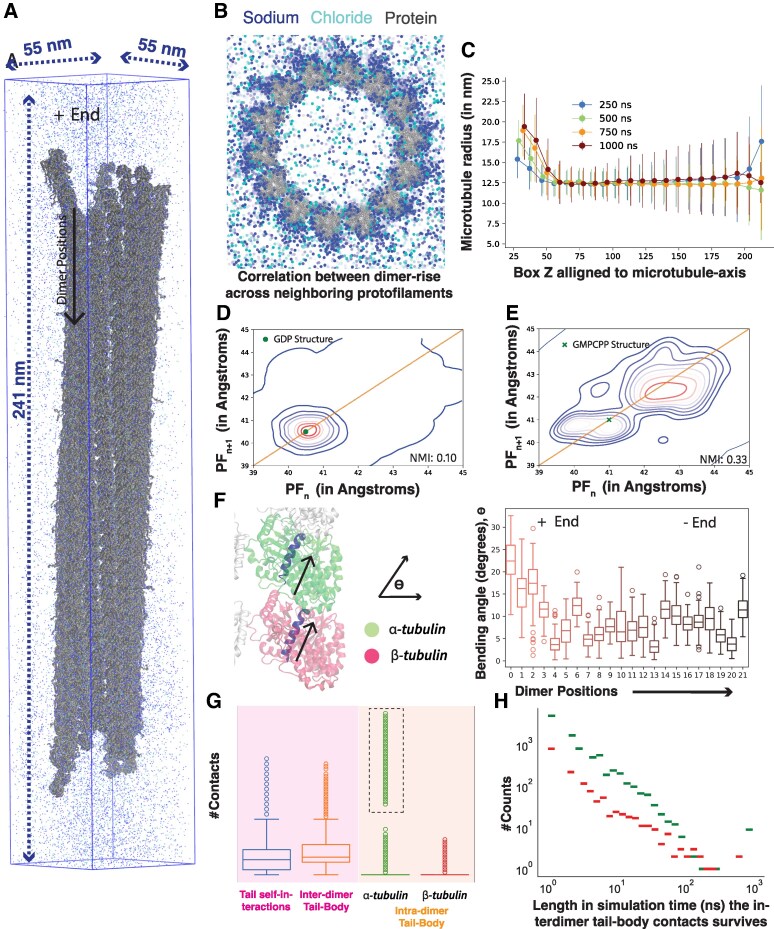
Structural analysis of 200 nm microtubule simulations. A) Representative snapshot of the whole microtubule, with ions shown for reference. B) Representative snapshot of the microtubule plus end. C) Axial view of the microtubule with ions. D) Contour plot showing correlation between the interdimer distances of adjacent protofilaments. E) Density distribution of sodium ions from the microtubule axis. F) Angle made by regression lines representing H7-helix of *α* and *β*-tubulin over microtubule length for a central protofilament. G) The types and number of contacts formed by the disordered C-terminal tails. H) The length of simulation-time (in ns) a contact between the C-terminal tail and the neighboring tubulin dimers survives for *α* and *β* tubulins.

In this section, we highlight some preliminary structural observations from our simulations of GDP-bound microtubules (∼200 nm long, 25 tubulin–dimers, ∼8 million interaction-centers; Fig. 4A and B). For these analyses, we have aggregated the trajectory data from three independent replicas, each run for 1μs. The simulations successfully replicated local bending phenomena, at the extremities of microtubules—driven by thermal fluctuations, whilst preserving the microtubule’s intrinsic cylindrical form (Fig. 4A and B; Supplementary material S2, Supplementary Video S1).

Particularly, our simulations could capture the peeling off of the microtubule from the plus end over simulation time as demonstrated by the local radius of the microtubule, measured by the distance of tubulin backbone sites from the microtubule axis (Fig. 4C). Fluctuations are higher at the minus end pointing to a wider range of arrangement of individual protofilaments—both inwards and outwards with-respect-to the microtubule lattice. The increase in radius is more sustained at the plus end, with lower errorbars, suggesting a dominantly radially outward dynamics.

Similar to the observations by Igaev et al. (19), our study also identified a correlation in the dimer separation (*α–β*) across neighboring protofilaments of the microtubule. However this effect was varied across protofilament number and time, depending on whether it forms contacts across protofilaments (Fig. 4D and E). This microscale interaction could be pivotal for influencing alterations in the lattice configuration and modulating the localized interplay between the microtubule and its binding proteins, including motor proteins, which depend on the spacing within the lattice structure for their functions (61). Moreover, this could also speak to the relative stabilities of different nucleotide associated states or tubulin isoforms and how perturbations in lattice travel along the microtubule. Here, we observed this correlation for both GDP (Normalized mutual information score—0.11, Pearson’s correlation—0.36) and GMPCPP lattice (Normalized mutual information score—0.33, Pearson’s correlation—0.57). Although this is particularly enhanced for GMPCPP lattice, possibly because of their location on the microtubule tip, allowing more thermal destabilization. Additionally, peeling off of the microtubules from the plus end always features multiple protofilaments in contact (Fig. S4), which could contribute to this enhanced correlation.

Given the explicit-solvent nature of our simulations, we can also report local enrichment of solvated ions. Figure S5 is the normalized probability of distances of ions measured from the microtubule axis. We observed an increased partitioning of monovalent ions to densities greater than 5 times the bulk-density at the microtubule surface. Particularly, the density of ions partitioned at the outer-side was higher than the inner. This could be due to highly charged disordered C-terminal tails on the tubulins that contribute to local accumulation of ions. Moreover, we observe an ion screening distance (debye-length) of close to 1.5 nm, similar to previous estimates from experiment (62).

From a microscopic perspective of dimer structure, we calculated the bending/curving of individual dimer through the angle made by the H7-helix of *α* and *β*-tubulins (Fig. 4F). H7-helix is a conserved core helix of tubulins that has contacts with the nucleotide-binding domains, and has been previously used to characterize dimer bending (63). Figure 4F shows the distribution of the angles of a nonseam protofilament (all protofilaments—Figs. S6 and S7) and the microtubule seam (Fig. S8), respectively. In both cases, an enhanced bending at the microtubule termini, particularly the plus end was observed, which can be likely attributed to the reduced stabilizing interactions between adjacent protofilaments and tubulins at these ends. The large error-bars suggest significant conformational dynamics of tubulin dimers irrespective of their position on the microtubule—seam, nonseam, tips or interior-lattice, over the simulation time. Another effective indicator of protofilament bending can be the angle between tubulin dimer center-of-mass (Figs. S9–S11). Here, we also notice increased angles closer to the microtubule tips—particularly the plus end—suggesting a complex interplay of both tubulin dynamics (intrinsic tubulin dimer curvature) and interaction between tubulin dimers. Please note here that interactions between adjoining protofilaments can contribute to multiple protofilaments peeling off together (Fig. S4, Supplementary Videos S1 and S2), as noted from prior all-atom simulations of microtubule tips (20).

Finally, we have characterized the types of interactions that the disordered C-terminal tail makes with the rest of the microtubule (Fig. 4G and H). Here, the residues were defined as interacting if residues more than two contiguous groups apart were within 7 Å of each other. We categorized this by the self-interactions of the tubulin tails, tail–body interactions within a dimer, and tail–body interactions across dimers. While highly dynamic, we observed that the C-terminal tail forms frequent contacts with itself and the microtubule body. Particularly, it can form contacts with neighboring dimers on the same protofilament, as demonstrated by the long-tailed distribution in Fig. 4G. Once formed, these contacts can survive long coarse-grained simulation times sometimes persisting up to the entire length of the simulation, thereby suggesting a stabilizing interaction. This observation supports the hypothesis initially posed by the NMR studies by Wall et al. (48) that the C-terminal tails can provide additional stabilizing interactions to aid and modulate microtubule dynamics. Moreover, our simulations show that these interactions are primarily driven by *α*-tubulin’s C-terminal tails interacting with the *β*-tubulin of the neighboring dimer towards the minus direction along a single protofilament. This work contributes to the growing body of research highlighting the importance of C-terminal tails at modulating various stages of microtubule stability and dynamics (46, 64, 65).

These preliminary observations suggest a microtubule lattice that reproduces local structural and dynamic patterns previously reported in literature.

## Discussion and conclusions

In this work, we introduce a Martini 3.0 microtubule capable of stably producing a 1μs simulation of a ∼200 nm GDP-bound microtubule with GMPCPP cap. This choice provides the ability to not only easily add other proteins to the simulation box but includes the residue level detail required for simulation of explicit solvent around the microtubule and the highly charged disordered C-terminal tails. Here, the elastic network is iteratively parametrized to reproduce distance fluctuations in atomistic simulations of microtubule patches. Our methodology employs a parametrization strategy originally introduced by Globisch et al. (56) and maintains fidelity to the original top-down parameterized Martini Hamiltonian while introducing modifications to the force field via the heterogeneous elastic network, which is effectively a correction term, resulting in a hybrid forcefield. Therefore, this model leverages all the benefits afforded by the Martini forcefield such as a amino acid sidechain level specificity, large library of parametrized interaction partners (proteins, small molecules, lipids, and metabolites), and easy optimization/modification (50, 50, 66, 67). To note, the current absence of validated Martini 3 parameters for key nucleotides, combined with its known limitations in handling highly charged moieties (for example artificial aggregation or “stickiness”), motivates our use of a nucleotide-agnostic elastic network model to bypass the need for direct parameterization. This approach differs from primarily bottom-up elastic network models like hENM (68), which are well-suited for capturing large-scale fluctuation modes and emergent elasticity in simplified lattice systems, but lack the chemical specificity needed to model many biologically relevant processes.

With these alterations we are able to create stable simulations of capped microtubules in explicit solvent. Beyond methodological contributions, our molecular simulations reveal some key structural features of microtubules. We observed a significant correlation between the neighboring protofilaments, particularly at the GMPCPP tips, where the tubulins assumed more expanded and bent conformations due to thermal fluctuations. Moreover, with our bending and axial compression simulations, we could reproduce close-to-experiment flexural rigidity, highlighting the robustness of the computational framework. By comparing both these approaches, we also demonstrate the effects of our assumptions (of the mechanical model of microtubules) on this computation. In this work, through controlled in silico experiments, we also identified a progressive increase in bending rigidity with the sequential addition of capping GMPCPP-bound tubulin dimers. These molecular insights deepen our understanding of the relationship between tubulin conformations, nucleotide states, and the emergent mechanical properties of microtubules.

Through our simulations, we have also demonstrated that the C-terminal tails of tubulin engage in dynamic interactions with the neighboring tubulin dimers. While similar interactions were proposed in earlier NMR studies by Wall et al. (48), our work provides a detailed and microscopic perspective. These interactions are primarily driven by the acidic residues of the *α*-tubulin tails, which interact with basic residues in the *β*-tubulin bodies of adjacent heterodimers, across a single protofilament in the minus direction of the microtubule. These disordered tails are particularly vulnerable to point mutations and posttranslational modifications such as phosphorylation, acetylation, and polyglutamylation (46), however given their disordered nature it is difficult to discern the effect of such modifications via experimental methods. Our model provides a potential avenue to investigate these effects and the emergent local, global, and mechanical behavior from a molecular perspective, potentially contributing to neurodegenerative diseases and other pathologies (69, 70).

Recent light microscopy and cryo-ET work, coupled with mathematical modeling on female meiotic spindles of *C. elegans* have reported microtubules at length scales close to those used in this work (71). While this can serve as a validation for our chosen length scales for microtubule simulations, particularly in applications to spindle-like systems, we acknowledge that the sizes are below the expected ranges for mammals. Here, we want to reiterate that simulations reported in this work primarily serve as a demonstration of the reported methods and future work can easily modify these relative sizes depending on the biological context.

The Martini forcefield has been extensively adopted by the research community to accurately study macromolecular systems, with diverse components at large spatiotemporal scales, ranging from complete virions, ∼120 MDa nuclear pore complex to proteins in crowded cell-like environments (38, 72–74). A common feature in these large systems is how microscale, local interactions compound, resulting in macroscale and/or mechanical changes—bridging information across multiple scales. Therefore, supplementing this popular forcefield with an appropriately parametrized heterogeneous elastic network that encodes local microscale interactions while preserving macroscale features, addresses an important challenge in multiscale modeling. Therefore IDEN and other similar and derivative methods could be potentially useful in capturing realistic dynamics of protein assemblies, with local lattice-like architecture, within the broader Martini framework.

In the context of microtubules, along the similar vein as this work, Martini enhanced with IDEN also makes it possible to study other nucleotide associated states and tubulin isoforms/constructs at larger spatiotemporal scales (75–77). This approach is particularly helpful in alleviating some key drawbacks of the Martini model regarding accuracies of nucleotide and/or small-molecule forcefield by implicitly encoding their effect in form of local fluctuations. This also enables understanding the diffusion and binding of several microtubule-associated proteins (such as Tau, PRC1, and EB1) and other small molecules like taxol on microtubule lattice (78–84). For example, tau—an important microtubule binding protein associated with the Alzheimer’s disease—has been recently shown to form condensates while interacting with the microtubule, thereby rearranging local dimer separation, which can in turn rearrange neighboring protofilament architecture, as evidenced from correlated dimer-separations on adjoining protofilaments in Fig. 4D (78). Another potential area for future research could involve assessing the influence of changing salt concentrations on microtubule dynamics. In our research, we observed localized accumulation of sodium ions in the outer regions of the microtubule, especially near the tubulin C-terminal tails. Previous research has documented the effects of varying salt concentrations on microtubule bundling and interactions between microtubules (11). Employing an explicit-solvent model capable of capturing local salt concentration fluctuations, this coarse-grained microtubule model could significantly contribute to advancing our understanding in those areas. More broadly, this framework opens avenues for exploring a range of microtubule-mediated phenomena—including protein diffusion along the lattice, membrane interactions, binding-induced changes to microtubule mechanics, lumenal transport, and environment-sensitive behaviors such as crowding-induced clustering or salt-dependent bundling—across diverse structural and biochemical contexts.

We acknowledge some key limitations in the parametrization process. The heterogeneous elastic network parametrization involves solving a multidimensional optimization problem. In this work, we addressed it using a simple regression approach, which inherently limits the likelihood of achieving robust convergence. This limitation arises from the computational cost, as each iterative step involves ∼1 μs of molecular simulation, making optimization through other methods that often involve several steps, computationally prohibitive. Moreover, the presence of the base Martini forcefield itself might prevent us from accessing the exact normal mode fluctuations in the atomistic forcefields. We also note here that there are other update rules suggested in the literature for iteratively updating spring constants for elastic network models, such as the ones developed in Lyman and Voth (68). While both methods could in principle be used for optimization, in this work, we used the direct update rule as stated in Globisch et al, due to faster convergence as noted in their work (56).

Since this model builds on the massively popular Martini model to derive its benefits, it also inherits Martini’s problems (54, 85–89). Even with significant reparameterizations in the newest iteration of the forcefield, there are still reported problems with protein–protein interactions (85, 89). This is particularly relevant for disordered regions. As an important functional region of the microtubule is disordered—C-terminal tails, this inability to capture accurate conformational space is an important limitation. While, in this work we have been particularly careful in reproducing this space to the accuracies comparable to AA simulations through re-scaling of protein–water interactions, Martini 3 still generated a somewhat compacted conformation evidenced by increased self-interactions within each tail. Moreover, even with a significantly reduced elastic network model to allow for more fluctuations, this model cannot still capture large conformational transitions such as folding/unfolding of domains. Another important limitation of the forcefield is the inaccurate entropy–enthalpy balance. Considering the optimization strategy employed for Martini forcefields involves balancing interactions between coarse-grained interaction centers to reproduce accurate free energies, this can lead to inaccurate entropy–enthalpy balance. Therefore, we need to be careful in considering kinetic information, and primarily focus on comparisons rather than absolute kinetics. Finally, with classical molecular dynamics forcefield, it is not possible to capture active processes that are often associated with the microtubules such as GTP hydrolysis and walking of motors without careful additional modifications. Beyond the forcefield, another challenge in this approach was appropriately modeling the microtubule seam. Here, we inferred the structure by rotations and translations from the cryo-EM lattice. Therefore, we do not accommodate for any conformational variances or specific geometry at the seam, considering unavailability of structural data. The Martini force field, while widely used, suffers from limited transferability due to its top-down parameterization against specific thermodynamic conditions. This can lead to artifacts such as inaccurate phase behavior under conditions different from those used during parametrization, stemming from the absence of explicit many-body or environment-sensitive terms. However, Martini performs well across chemical space for molecules such as a wide range of lipids, small molecules, and surfactants. This highlights a key trade-off: while Martini captures chemical specificity effectively, its lack of thermodynamic adaptability limits its generalizability. Strategies to enhance transferability could include using bottom-up approaches and/or machine learning-based potentials that incorporate multistate training or environmental descriptors (90–92).

Future work in our group aims to address some of the limitations—particularly simple structural transitions and some reactive processes with alternatives of elastic-network that allow spontaneous forming and breaking of bonds (66, 93–98). A recently developed proxy for elastic network are Gō-like models that encode conformational dynamics more effectively, similar to Gō-Martini (99, 100). This will possibly help in capturing more disordered states at microtubule tips, and binding–unbinding dynamics of tubulins. Furthermore, this will build a stronger bridge with thermodynamics through carefully parametrizing the Gō model to reproduce experimental binding affinities. A carefully parameterized Gō model—guided by steered MD simulations or single-molecule pulling experiments—may also allow us to capture large-scale transitions such as catastrophe events. Furthermore, implementing dual-basin Gō potentials offers a promising route to model conformational switching within the lattice, particularly in scenarios where microtubule-associated proteins induce local lattice destabilization. These hybrid simulations could therefore be leveraged to explore protein-induced remodeling and heterogeneity across the microtubule.

To summarize, this work presents a first Martini microtubule, with all the benefits and drawbacks of that choice of forcefield, as well as the scheme used to make this microtubule stable. We believe this will provide a valuable stepping stone for other CG-MD simulations seeking to characterize the molecular biophysics dictating microtubule function, including but not limited to solvent effects, protein binding partners, and microtubule lattice dynamics. Finally, this approach to microtubule molecular dynamics can also be used in tandem with cryo-EM to understand the intrinsic dynamics of microtubules and their binding partners seen in various relevant and well-studied in vitro and in vivo environments (101, 102).

## Materials and methods

### CG simulation protocol

Sodium chloride at 150 mM concentration was introduced to solvated protein systems, followed by energy minimization via steepest descent until achieving machine precision. Subsequently, an initial equilibration phase of 100 ns (timestep: 2 fs) was undertaken in the isothermal ensemble, with protein backbone beads restrained (force constants: 1,000 kJ/mol). Three additional equilibration steps for 100 ns each followed, step-wise increasing timesteps (2 fs, 5 fs, and 10 fs) in the isothermal-isobaric ensemble, while keeping the protein backbone restrained. Throughout equilibration, a v-rescale thermostat and Berendsen barostat maintained constant temperature (300 K) and pressure (1 bar). Ultimately, a 1 μs production simulation with a 10 fs timestep, Parinello-Rahman barostat, coupling time constant of 12 fs, and compressibility of $3.0\times 10^{-4}$/bar was run (103). For all the equilibration, and production steps, the electrostatic cutoff distance is set at 1.6 nm, and to account for long-range electrostatic interactions, we employ the particle-mesh Ewald (PME) method (104, 105).

### All-atom simulation protocol

All the all-atom simulations were performed with gromacs 2022.4 with CHARMM36m/TIP3P forcefield (106, 107). The initial model of a microtubule patch was downloaded from the cryo-EM structures deposited in the protein data bank—6DPV for GDP microtubule, 6DPU for GMPCPP cap (42). The microtubule patches are elongated along the microtubule axis, to twice the length of deposited structures. The C-terminal tails and other smaller internal missing loops were added to the structure with CHARMM-GUI (108). The initial structure was passed through the CHARMM-GUI solution builder to generate the input files for the simulation. Water and 150 mM sodium chloride is added to the simulation system, before energy minimization with stochastic gradient descent to reach machine precision. The system was equilibrated for 1 ns with isothermal, isochoric ensemble using v-rescale thermostat at a coupling time of 1 ps. Following this, the system was further equilibrated in a isothermal, isobaric ensemble for 5 ns. For all the equilibration steps, the protein backbone is restrained allowing for side-chain and solvent to equilibrate.

In our production simulations, strategic position restraints were applied to distinct microtubule segments, allowing us to focus on specific regions of interest. Within the microtubule lattice, we imposed position restraints on the backbone of the two terminal tubulin monomers, with a force constant of 10 kJ/mol. The tubulin contacts situated farthest from the restrained tubulins were employed to derive the corresponding elastic network.

### Iteratively refined distance-based elastic network

Our approach for refining ENM involved first defining a list of contacts $D=d_{ij}$, the distance between nonneighboring C-*α* atoms in the PDB structure, was within 5–9 Å ($L_{\mathrm{cutoff}}$), similar to the original recommendations by the Martini forcefield (54, 55). This list was further sub-sampled by removing outliers, where the experimental values from the PDB-derived structures are outside three standard deviation of the corresponding probability distribution—derived from the atomistic simulations of the patch, assuming a local normal distribution. This sub-sampling explicitly removes the elastic network connections from highly dynamic parts of this biomolecular assembly.


(1) $$\begin{array}{c} D\;:\;\left\{d_{ij}|\begin{array}{c} \left|i-j\right|>2,\\ d_{ij}\in \left[0.5\mathrm{nm},0.9\mathrm{nm}\right],\\ \frac{d_{ij}-\mu \left(d_{ij}\right)}{\sigma (d_{ij})}\;<3 \end{array}\right\} \end{array}$$

The elastic network strength ($k_{ij}$) is initially constructed inversely proportional to the variance of respective distances ($d_{ij}$), resulting in a heterogeneous elastic-network rather than uniform elastic-network often used in Martini simulations.


(2) $$k_{i,j}=k_{0}\;\frac{\min(\sigma_{ij}^{2})}{\sigma_{ij}^{2}}$$

where, $\sigma^{2}$ refers to the variance of contact distances; and $k_{0}=500$ kJ/mol.

The initial atomistic model is then coarse-grained using the Martini 3 mapping scheme. Following minimization and equilibration, the production simulation is run for 1 μs with position restraints on the end tubulins to mimic the atomistic simulations. The generated trajectory can now be processed to infer the elastic-network part of the forcefield. We have followed the iterative optimization strategy originally introduced by Globisch et al. to match the variance of coarse-grained distances to the all-atom simulations with the following update rule (56).


(3) $$k_{ij,n+1}=k_{ij}-\frac{\alpha k_{b}T}{L_{\mathrm{cutoff}}^{4}}\;(\sigma_{ij,AA}^{2}-\sigma_{ij,CG}^{2})$$

where, *n* refers to the iteration number, $\sigma_{ij,AA/CG}^{2}$ is the variance in contact-distances, *α* is a constant and in this work we have used 1050, similar to Globisch et al. (56). For the iterations, we tracked $D=\langle \sigma_{ij,AA}^{2}-\sigma_{ij,CG}^{2}\rangle$ over the last 500 ns of simulation time. The optimization was stopped when the when we reach convergence as outlined in the SI (Fig. 2C).

### Microtubules under axial pressure

To compute the flexural rigidity of our model microtubules, we followed closely the protocol outlined by Igaev et al. (19). First, we created a smaller microtubule (∼90 nm), and modified the box such that the microtubule is periodic along the axis (Z). The system was solvated and 150 mM ions were added. The initial equilibration followed the exact same protocol as for our other coarse-grained simulations, but with anisotropic pressure coupling ($P_{x}=P_{y}\neq P_{z}$). Finally, an additional long equilibration simulation was performed to equilibrate the simulation box along the microtubule axis, due to the additional axial force. The applied axial pressure, $P_{z}$ ranged from −0.25 bar to +2 bar. With values greater than +2 bar, the cylindrical architecture of the microtubule started falling apart. And at values below −0.25 bar, the microtubule was not able to maintain periodic contact, due to the elastic network. We acknowledge that going away from an elastic network model towards a Gō-like model here could be more accurate because that would allow us to then simulate microtubules at much higher values of extension and compression.

The net stress, $\sigma_{zz}$ can be computed as—


(4) $$\frac{(P_{zz}-P_{n})L_{x}L_{y}}{A_{z}}$$

where, $L_{x}$ and $L_{y}$ are box dimensions; $P_{zz}$, $P_{n}$ is the measured axial and normal pressure, and $A_{z}$ is the cross-sectional area of the microtubule. We calculated $A_{z}=\pi (r_{o}^{2}-r_{i}^{2})$ and the second moment of cross-sectional area—$I=\frac{\pi }{4}(r_{o}^{4}-r_{i}^{4})$, by first inferring the inner ($r_{i}$), and outer ($r_{o}$) radius of the microtubule using the inflection points in the distribution of protein-backbone distances measured from the microtubule axis.

The strain is computed as,


(5) $$\epsilon_{zz}=\;\frac{L_{z}-L_{z,\mathrm{eq}}}{L_{z,\mathrm{eq}}}$$

where $L_{z,\mathrm{eq}}$ is the mean axial box length.

The last 2 μs of simulation-time was used for inference, with block averaging through 200 ns windows for error analysis. This analysis used a net sampling time of ∼8μs.

### Steered molecular dynamics for bending microtubules

We also computed the flexural rigidity by using steered molecular dynamics simulations. First, the microtubules are equilibrated by position restraining the microtubule at the minus end. Following multistep equilibration as described in the coarse-grained molecular dynamics protocol section, we ran a longer final equilibration of 50 ns. Finally, we simulated pulling the center-of-mass of tubulins at the plus-end perpendicular to the microtubule-axis at a velocity 1 nm per 1 ns, till the maximum deflection of 15 nm. The flexural rigidity is calculated from the Euler–Bernouli beam theory for a clamped beam with load on one end.


(6) $$w=\;\frac{FL^{3}}{3EI},$$

where *w* is the deflection at the microtubule tip, *F* is the applied force, *L* is the length of the microtubule, *E* is the young’s modulus, and *I* is the moment of inertia. The product of young’s modulus and the moment of inertia is the estimated flexural rigidity (109).

All the simulations used in this work have been listed in the Table S1.

## Supplementary Material

pgaf202_Supplementary_Data

## Data Availability

All the scripts and forcefield files for Martini simulations with an iteratively refined distance-based elastic-network (IDEN) adapted for microtubules are shared here—https://github.com/flatironinstitute/martini-microtubule.

## Preprints

This manuscript was posted on a preprint: https://www.biorxiv.org/content/10.1101/2024.05.29.596440v1.
