# Supplementary material for: Microtubules in Martini: Parameterizing a heterogeneous elastic-network towards a mechanically accurate microtubule
Source: PNAS Nexus. 2025 Jun 21;4(7):pgaf202. doi: 10.1093/pnasnexus/pgaf202 (PMC12208291; doi:10.1093/pnasnexus/pgaf202)
Supplement: pgaf202_Supplementary_Data [file pgaf202_supplementary_data.zip › PNASNEXUS-PNASNEXUS-2024-01258RR-s01.pdf]

Supplementary Information for - Microtubules in Martini: Parameterizing a Heterogeneous Elastic-Network Towards a Mechanically Accurate Microtubule

Abhilash Sahoo [*1,2] and Sonya M. Hanson [†1,2]

[1]Center for Computational Biology, Flatiron Institute, New York, NY, USA

[2]Center for Computational Mathematics, Flatiron Institute, New York, NY, USA

*asahoo@flatironinstitute.org

†shanson@flatironinstitute.org

Type of simulations	Time	# of interaction sites (M=millions)
**Atomistic MD**		
GDP-patch	0.4 micro-seconds	4M
GMPCPP-patch	0.4 micro-seconds	4M
**Coarse-Grained MD**		
GDP tubulin lattice + ∼3 dimer GMPCPP cap	3 X 1 micro-seconds	6M
Axial-compression GDP tubulin lattice @ Pzz = -0.25 bar	2 micro-seconds	2M
Axial-compression GDP tubulin lattice @ Pzz =1 bar	2 micro-seconds	2M
Axial-compression GDP tubulin lattice @ Pzz =1.125 bar	2 micro-seconds	2M
Axial-compression GDP tubulin lattice @ Pzz =1.25 bar	2 micro-seconds	2M
Axial-compression GDP tubulin lattice @ Pzz =1.5 bar	2 micro-seconds	2M
Axial-compression GDP tubulin lattice @ Pzz =1.75 bar	2 micro-seconds	2M
Axial-compression GDP tubulin lattice @ Pzz =2 bar	2 micro-seconds	2M
Bending simulations - GDP tubulin only lattice @ 1nm/ns, 2nm/ns	upto deflection =15 nm	13M
Bending simulations - GDP tubulin lattice with 4 dimer GMPCPP cap @ 1nm/ns, 2nm/ns	upto deflection =15 nm	13M
Bending simulations - GDP tubulin lattice with 6 dimer GMPCPP cap @ 1nm/ns, 2nm/ns	upto deflection =15 nm	13M
Bending simulations - GDP tubulin lattice with 8 dimer GMPCPP cap @ 1nm/ns, 2nm/ns	upto deflection =15 nm	13M
Bending simulations - GDP tubulin lattice with 10 dimer GMPCPP cap @ 1nm/ns, 2nm/ns	upto deflection =15 nm	13M

Supplementary Table 1: List of all the molecular simulations run this work

Supplementary Figures

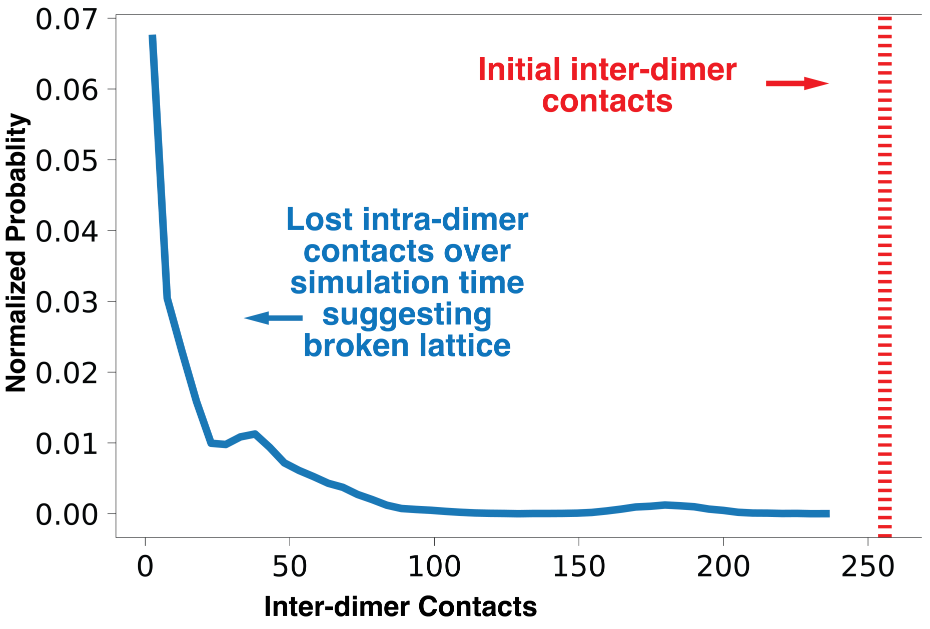

Supplementary Figure 1: The probability of inter-dimer contacts after 400 ns of simulation time, with no inter-dimer elastic network

1 Discussion on Convergence of Iteratively Optimized distance-based Elastic Network (IDEN)

In the case of heterogeneous elastic network models (hENM) and other similar methods, the optimization process is typically analytical or semi-analytical, relying on deterministic computations to minimize a loss function that quantifies deviations between target and predicted fluctuation modes.[1,2] Because of the analytical nature and the relatively smooth, well-conditioned loss landscape, it is common and often sufficient to define convergence using a fixed loss threshold. This works well since the expected scale of the loss can be reasonably anticipated and is consistent across runs, allowing practitioners to predefine a meaningful cutoff value (e.g., based on the number of modes or resolution). In contrast, our iterative framework integrates stochastic molecular simulations at each optimization step — for example, using ensemble-averaged quantities or noisy estimates of physical observables to evaluate the loss. As a result, the loss values can exhibit inherent fluctuations even when the underlying system has functionally converged. In such cases, a fixed loss threshold is often unreliable: it may lead to premature termination due to temporary noise suppression or, conversely, may prevent convergence from being recognized when the loss fluctuates slightly above the cutoff.

To address this, we adopt a variance-based convergence criterion, which assesses whether the loss values have stabilized by monitoring the variance over a sliding window. This allows us to detect convergence in a way that is both robust to stochastic noise and adaptive to the specific dynamics of the optimization process. Specifically, we monitor the variance of the loss values using a fixed-length sliding window—three and five steps in our case—to evaluate local fluctuations throughout the optimization trajectory. A consistent drop in this variance is used as a signal that the optimization process has stabilized. To set a convergence threshold in a data-driven way, we estimate a noise floor based on the median variance of the final three windows, where convergence is most likely to occur. This floor is then scaled by a tunable factor (1.5× in our analyses) to account for expected residual noise. Convergence is declared at the earliest point where the variance remains below this threshold for all subsequent windows. To increase the stringency of this criterion, we only accept convergence when both the three- and five-window analyses agree. Although convergence was typically detected earlier over the iterations, we used the forcefield from the final iteration for all downstream simulations.

This approach is motivated by principles from steady-state detection in molecular simulations ,[3] convergence diagnostics in Markov chain Monte Carlo sampling ,[4,5] and adaptive signal processing methods for change-point detection.[6,7] By leveraging a data-driven, system-specific threshold based on the intrinsic noise characteristics of the optimization, our method offers a robust and interpretable criterion for detecting convergence in iterative procedures where stochastic fluctuations are intrinsic to the loss evaluation.

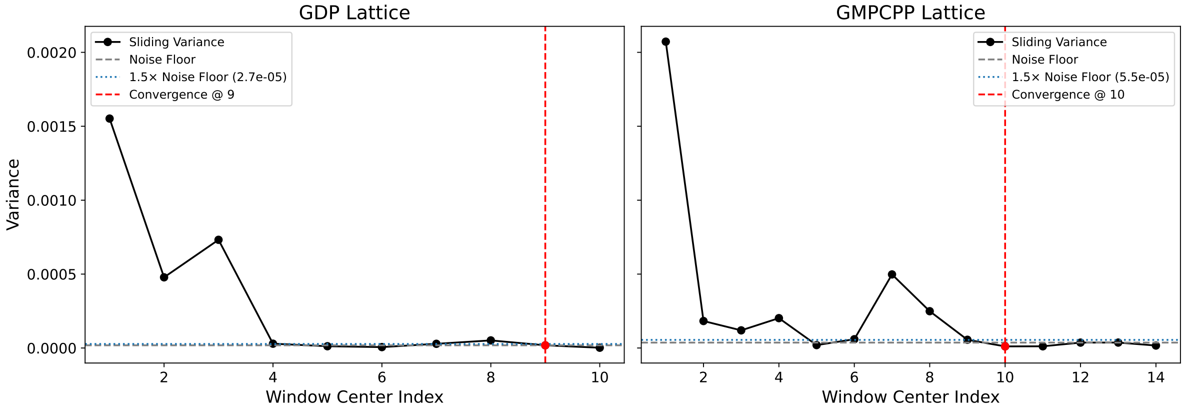

Supplementary Figure 2: Variance-based convergence detection applied to stepwise loss values for both lattice types. Loss variance is computed over a sliding window of **three** steps. A noise floor is estimated from the median of the last three windows, and convergence is defined as the earliest window after which variance remains consistently below a scaled threshold (1.5× the noise floor). Vertical dashed lines indicate detected convergence points, and horizontal dotted lines indicate the thresholds.

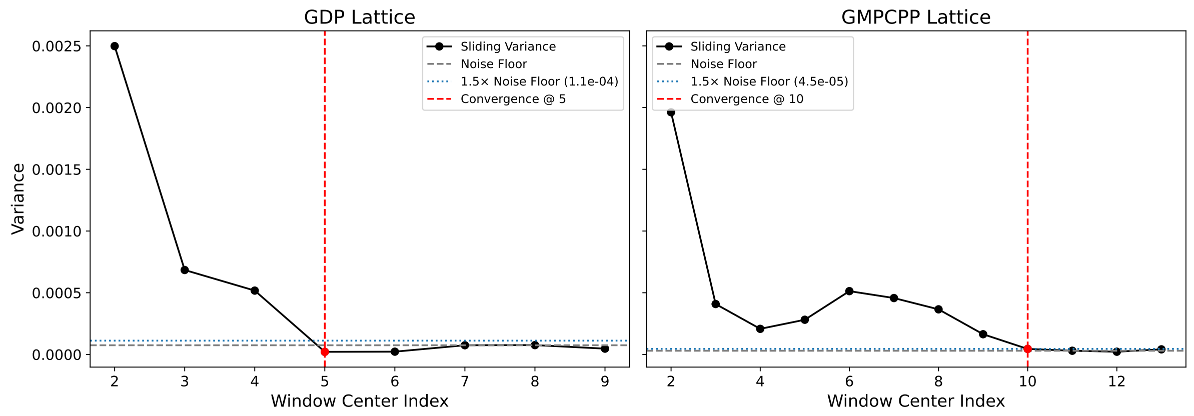

Supplementary Figure 3: Variance-based convergence detection applied to stepwise loss values for both lattice types. Loss variance is computed over a sliding window of **five** steps. A noise floor is estimated from the median of the last three windows, and convergence is defined as the earliest window after which variance remains consistently below a scaled threshold (1.5× the noise floor). Vertical dashed lines indicate detected convergence points, and horizontal dotted lines indicate the thresholds.

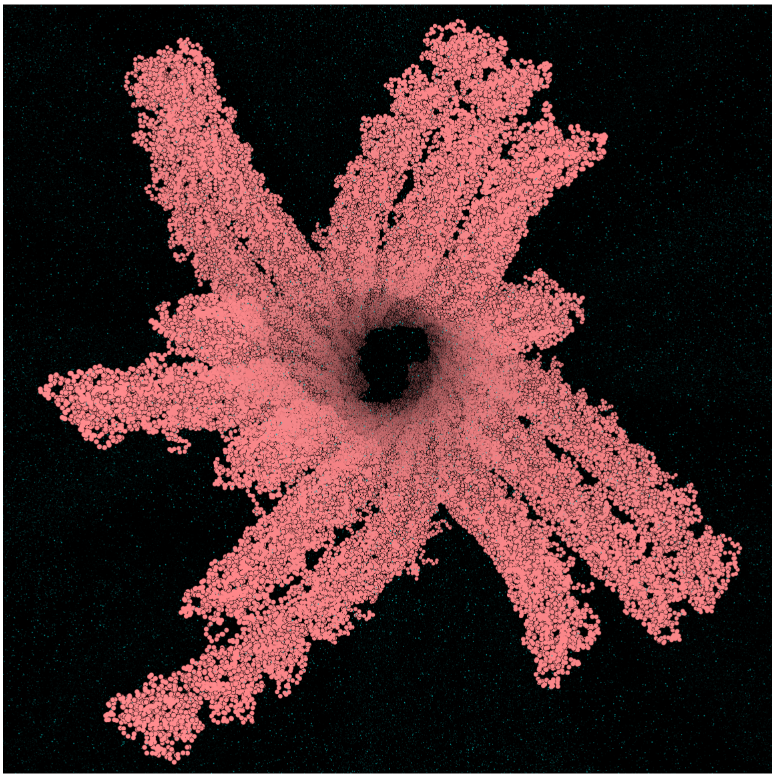

Supplementary Figure 4: Axial view of the microtubule from the plus end.

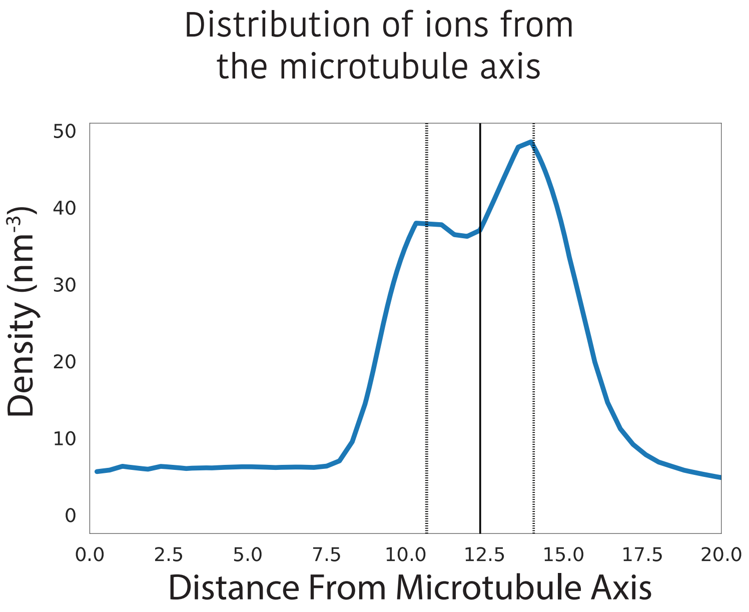

Supplementary Figure 5: Distribution of sodium ions from the microtubule-axis.

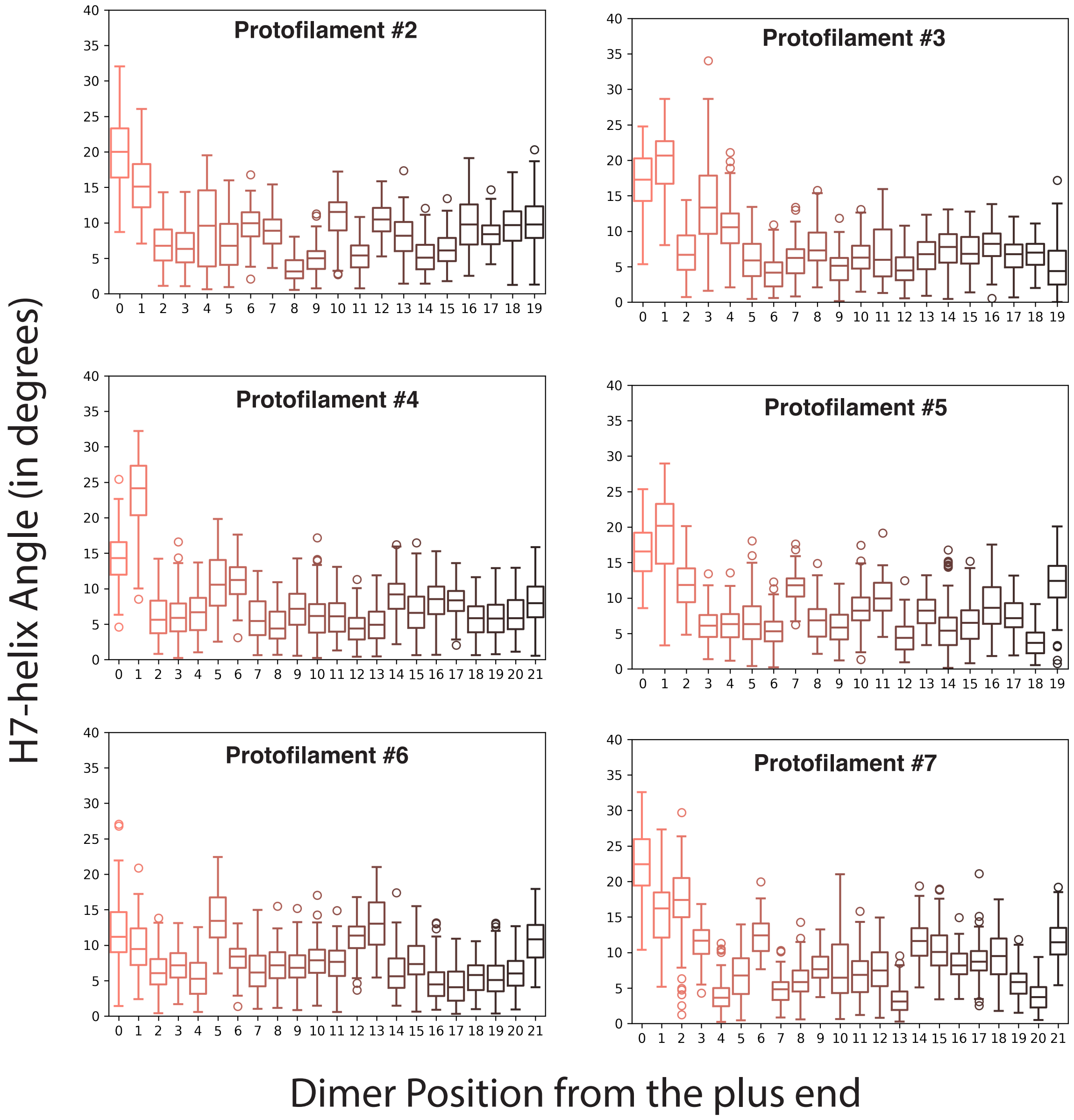

Supplementary Figure 6: Angle made by regression lines representing H7-helix of α and β tubulin over microtubule length for a non-seam protofilament

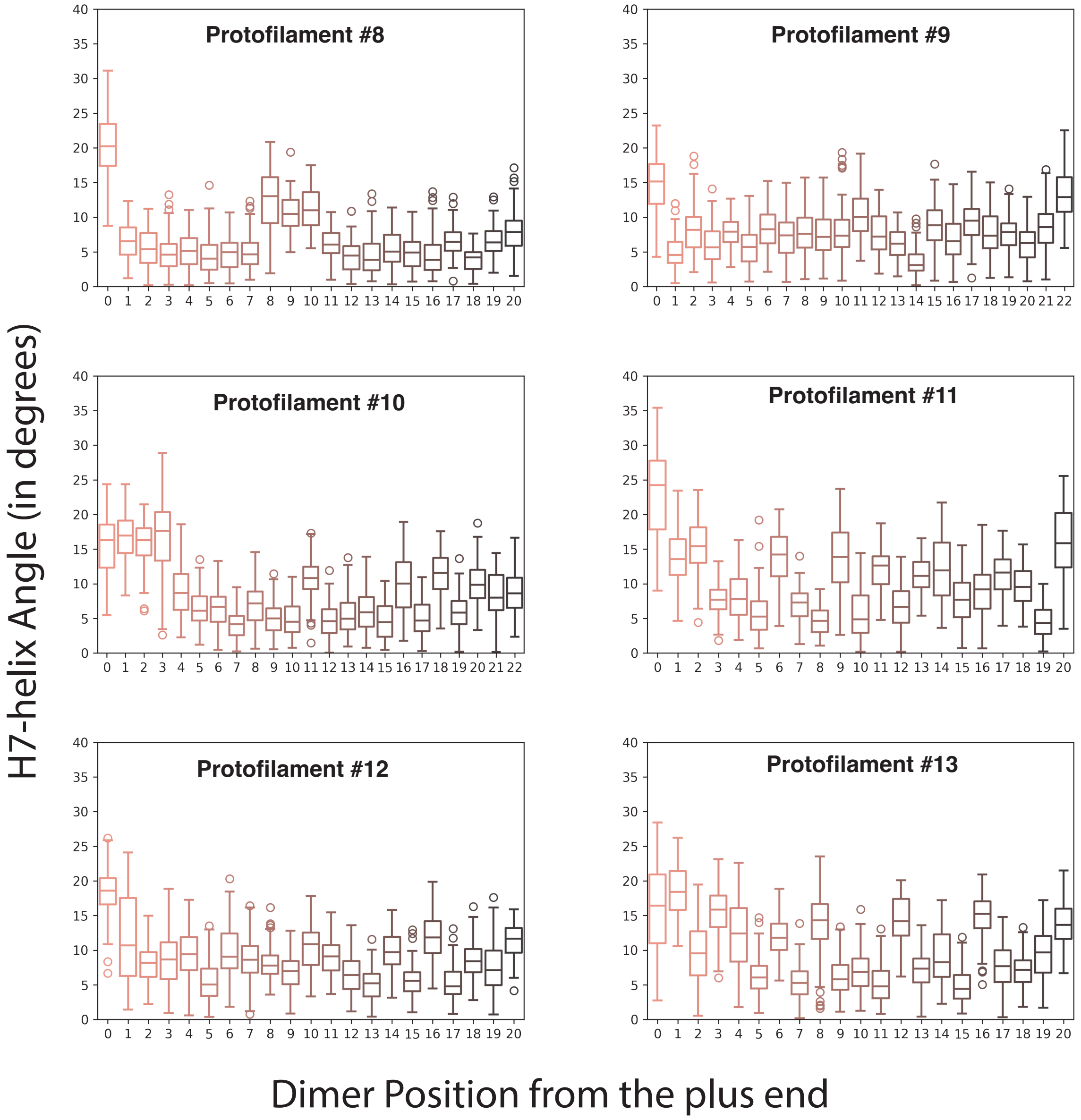

Supplementary Figure 7: Angle made by regression lines representing H7-helix of α and β tubulin over microtubule length for a non-seam protofilament

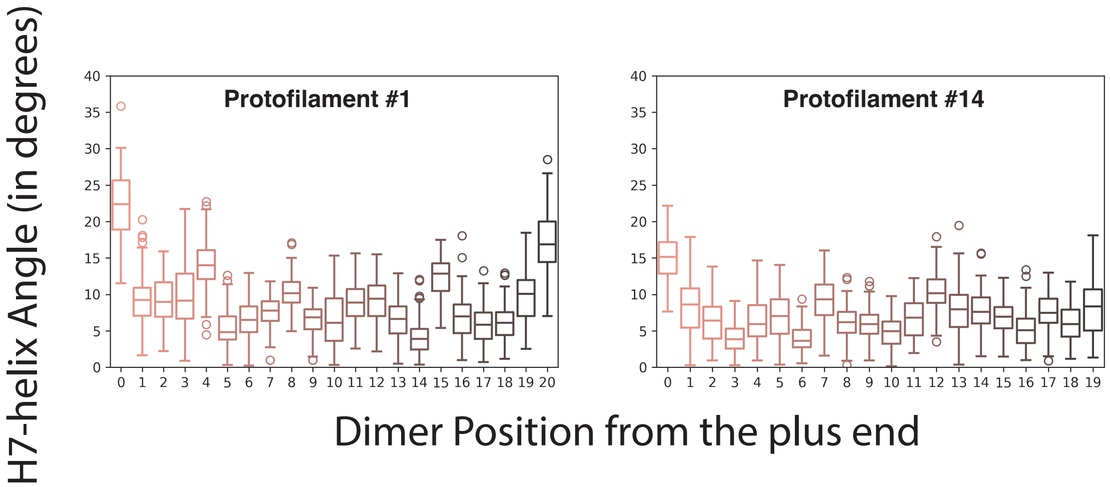

Supplementary Figure 8: Angle made by regression lines representing H7-helix of α and β tubulin over microtubule length for protofilament at the microtubule-seam

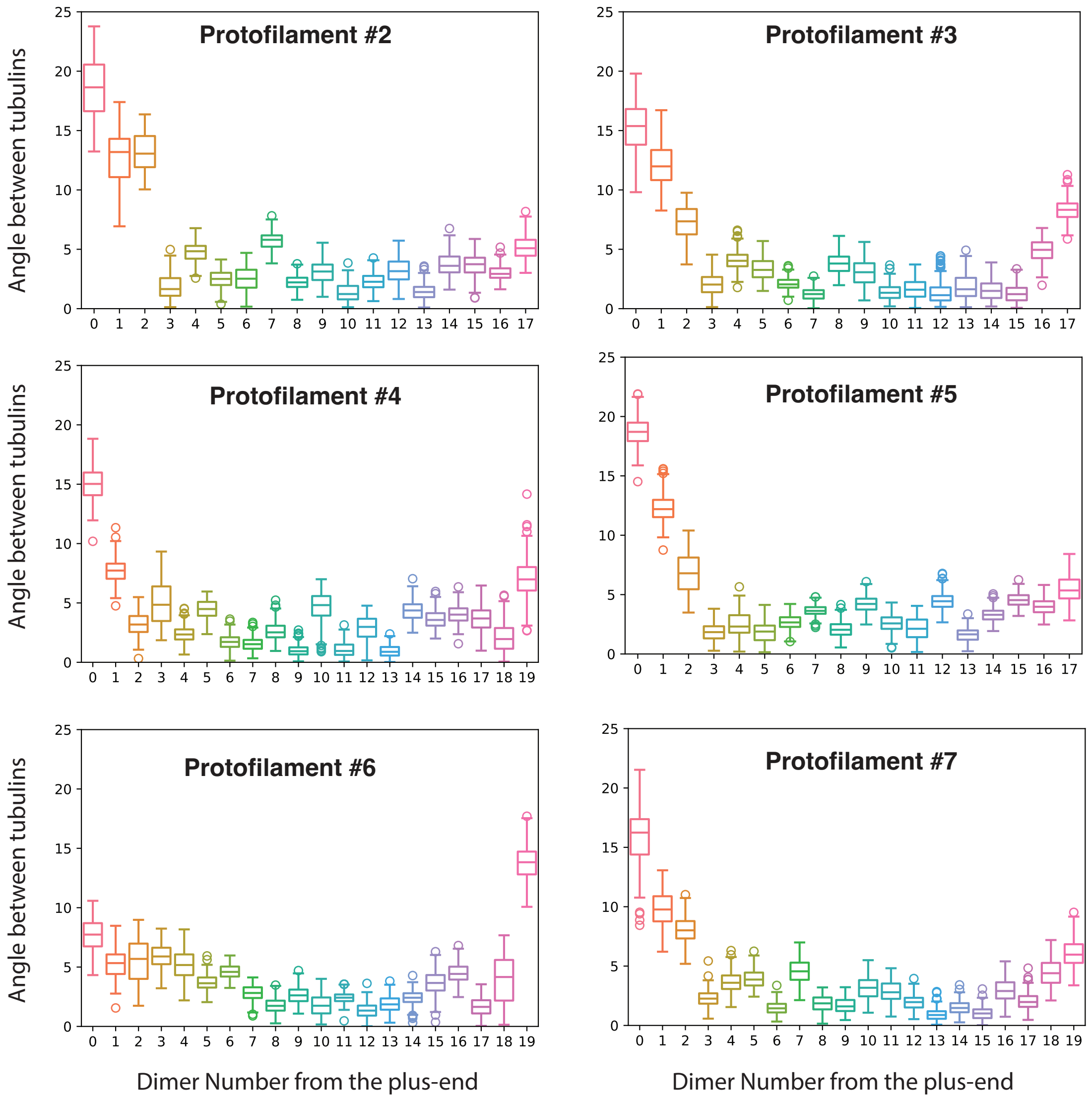

Supplementary Figure 9: Tubulin bending of the protofilament at the **non-seam** location, characterized by the angle between tubulin dimer center-of-mass

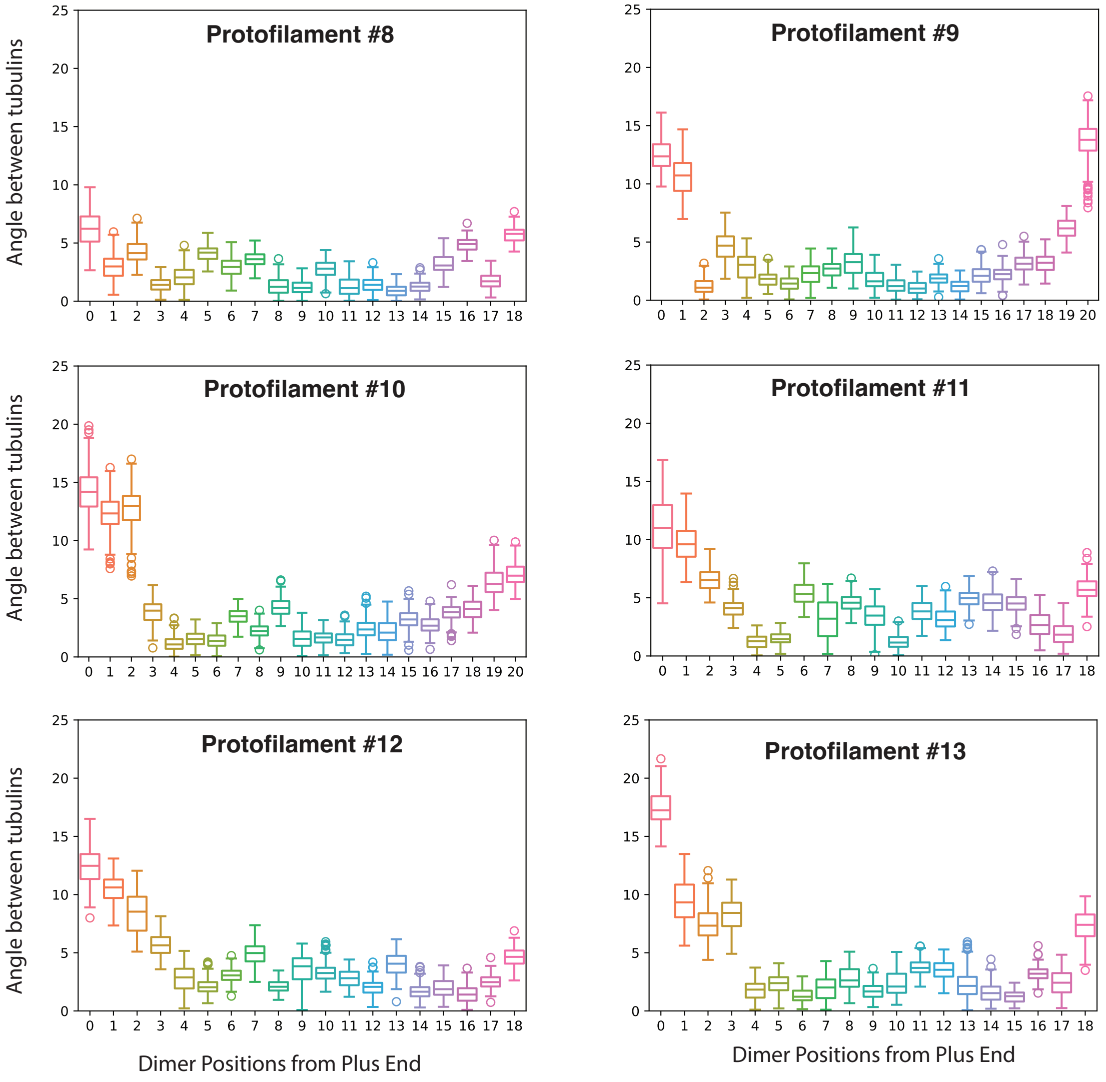

Supplementary Figure 10: Tubulin bending of the protofilament at the **non-seam** location, characterized by the angle between tubulin dimer center-of-mass

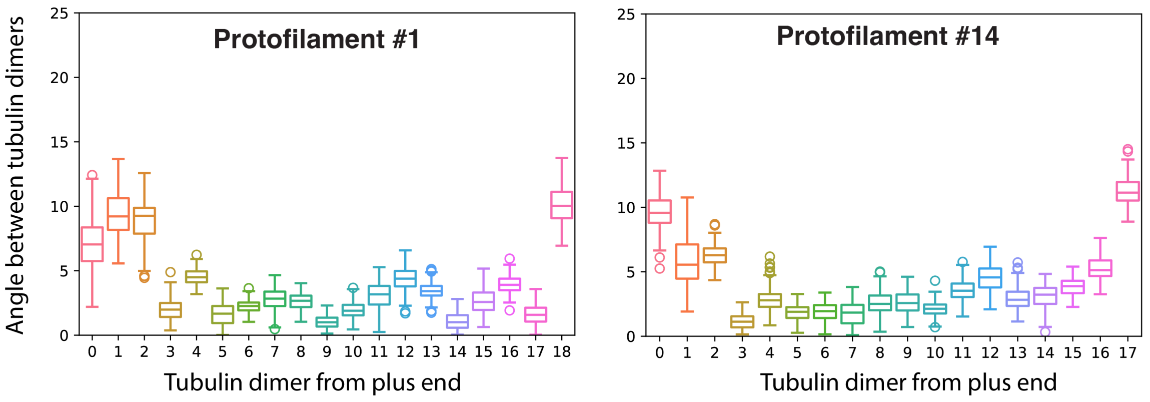

Supplementary Figure 11: Tubulin bending of the protofilament at the **seam**, characterized by the angle between tubulin dimer center-of-mass

Supplementary Video 1

1 micro-second trajectory of a full microtubule.

References

[1] Lyman, E.; Pfaendtner, J.; Voth, G. A. *Biophys. J.* **2008**, *95*, 4183–4192.

[2] Chu, J.-W.; Voth, G. A. *Biophys. J.* **2006**, *90*, 1572–1582.

[3] Chodera, J. D. *J. Chem. Theory Comput.* **2016**, *12*, 1799–1805.

[4] Gelman, A.; Rubin, D. B. *Stat. Sci.* **1992**, *7*, 457–472.

[5] Geweke, J. *Fed. Reserve Bank Minneapolis Staff Rep.* **1992**,

[6] Gustafsson, F. *Adaptive Filtering and Change Detection*; Wiley, 2000.

[7] Truong, C.; Oudre, L.; Vayatis, N. *Signal Process.* **2020**, *167*, 107299.
